# Bio-inspired hybrid microelectrodes: a hybrid solution to improve long-term performance of chronic intracortical implants

**DOI:** 10.3389/fneng.2014.00007

**Published:** 2014-04-10

**Authors:** Sara De Faveri, Emma Maggiolini, Ermanno Miele, Francesco De Angelis, Fabrizia Cesca, Fabio Benfenati, Luciano Fadiga

**Affiliations:** ^1^Department of Robotics, Brain and Cognitive Science, Istituto Italiano di TecnologiaGenova, Italy; ^2^Department of Neuroscience and Brain Technologies, Istituto Italiano di TecnologiaGenova, Italy; ^3^Department of Nanostructures, Istituto Italiano di TecnologiaGenova, Italy; ^4^Department of Experimental Medicine, University of GenovaGenova, Italy; ^5^Section of Human Physiology, University of FerraraFerrara, Italy

**Keywords:** gliosis, intracortical microelectrodes, fibrin, bio-inspired, *in-vivo*, chronic implants

## Abstract

The use of implants that allow chronic electrical stimulation and recording in the brain of human patients is currently limited by a series of events that cause the deterioration over time of both the electrode surface and the surrounding tissue. The main reason of failure is the tissue inflammatory reaction that eventually causes neuronal loss and glial encapsulation, resulting in a progressive increase of the electrode-electrolyte impedance. Here, we describe a new method to create bio-inspired electrodes to mimic the mechanical properties and biological composition of the host tissue. This combination has a great potential to increase the implant lifetime by reducing tissue reaction and improving electrical coupling. Our method implies coating the electrode with reprogrammed neural or glial cells encapsulated within a hydrogel layer. We chose fibrin as a hydrogel and primary hippocampal neurons or astrocytes from rat brain as cellular layer. We demonstrate that fibrin coating is highly biocompatible, forms uniform coatings of controllable thickness, does not alter the electrochemical properties of the microelectrode and allows good quality recordings. Moreover, it reduces the amount of host reactive astrocytes – over time – compared to a bare wire and is fully reabsorbed by the surrounding tissue within 7 days after implantation, avoiding the common problem of hydrogels swelling. Both astrocytes and neurons could be successfully grown onto the electrode surface within the fibrin hydrogel without altering the electrochemical properties of the microelectrode. This bio-hybrid device has therefore a good potential to improve the electrical integration at the neuron-electrode interface and support the long-term success of neural prostheses.

## INTRODUCTION

Patients with various neurological disorders could benefit from the stereotaxic implant of chronic intracerebral microelectrodes to record endogenous activity and/or to electrically stimulate specific brain areas (Donoghue et al., 2007; Larson, 2008; Truccolo et al., 2008; Velliste et al., 2008; Benabid et al., 2009; Normann et al., 2009; Hemm and Wårdell, 2010). To be functional and safe, the implant should keep a stable electric interface with the tissue, enabling an efficient recording and/or activation of neurons over time. However, the long-term functionality of implanted electrodes has been very critical, because of the progressive deterioration of both the electrode surface and the surrounding neural tissue.

Electrode insertion is a traumatic event that induces acute injury to the brain tissue (Polikov et al., 2005; Grill et al., 2009; Marin and Fernández, 2010). The tissue is cut, stretched or compressed by the penetration of the electrode and neurons and glial cells within and around the electrode path are killed or severely injured during insertion. Since the first day of implantation, microglial cells -that are extremely sensitive to even small pathological changes- start to be activated to scavenge damaged neurons and cell debris. Moreover, activated microglia also releases pro-inflammatory cytokines that propagate the inflammatory reaction. The deepening of the electrode into the brain pushes aside the tissue and increases the local pressure of the surrounding tissue. Blood vessels and capillaries are disrupted and the blood–brain barrier is compromised (Bjornsson et al., 2006), leading to plasma extravasation and local edema, that further increase the intracranial pressure (Unterberg et al., 2004).

This initial acute tissue reaction triggers, and is followed by, a more adverse long-term response mainly operated by activated astrocytes that is dependent on the extension of the injury and ends up in the generation of a glial scar, neuronal loss and impairment of the regrowth of neuronal processes (Fawcett and Asher, 1999; Polikov et al., 2005; Grill et al., 2009; Marin and Fernández, 2010). The glial encapsulation surrounding the electrode becomes denser and organized over time pushing away neurons from the tip of the probe (the so-called “kill zone”). This leads to a progressive increase of the electrode-electrolyte impedance and to a deterioration of the recordings. All these events reduce the long-term performance of the electrodes, making the long-term application of this type of technology to human subjects almost unfeasible.

The physical properties of the electrodes such as surface composition, size, shape and stiffness, the mechanical connection between the probe and the recording system, the functionalization of the electrode and the implantation method all have some impact on the intensity of the tissue reaction (Edell et al., 1992). Different approaches have been proposed to solve or reduce the inflammatory reaction. One is the reduction of the electrode size. Minimizing the device footprint is important to reduce the insertion trauma. A smaller device induces a much smaller increase in the local pressure and minimizes tissue displacement (Kozai et al., 2012). However, miniaturization of the electrode worsens its electrochemical characteristics in terms of contact impedance and signal-to-noise ratio (SNR). To circumvent this effect, various groups have used conductive polymers such as poly(3,4-ethylenedioxythiophene, PEDOT) or nanostructured materials (e.g., carbon nanotubes) to reduce the impedance of the recording site (Richardson-Burns et al., 2007; Kim et al., 2010b; Ansaldo et al., 2011; Baranauskas et al., 2011).

Other solutions employing untethered or flexible probes (Rousche et al., 2001; Takeuchi et al., 2005; Kim et al., 2010a; Castagnola et al., 2013; Zhang et al., 2013) have been adopted to decrease the formation of the glial scar. Indeed, when an electrode is tethered to the skull or the device has a different compliance with respect to the adjacent brain tissue, the brain, subject to normal movement induced by respiration and circulation, moves around the probe causing chronic trauma as well as instability of the recording (Kim et al., 2004b; Biran et al., 2007; Thelin et al., 2011).

Approaches aimed at reducing the mechanical mismatch at the interface between the stiff intracortical electrode and the soft brain tissue have been proposed. Intracortical wires were covered with soft materials (Kim et al., 2010b) or with polymeric nano-composite materials that switch from a stiff to a compliant state when inside the cerebral tissue (Harris et al., 2011). The soft coating, matching the host tissue softness, was shown to play an important role in limiting the severity of the inflammatory reaction over time in terms of the amount of reactive astrocytes, neuronal death, and density of active microglial cells around the probe (Harris et al., 2011). The use of hydrogel scaffolds has also been introduced to contrast the consequences due to the inflammatory and immune responses by astrocytes and oligodendrocytes involved in the assembly of the glial scar. Both biologically derived hydrogels (e.g., alginate, Frampton et al., 2011; hyaluronic acid, Hou et al., 2005) and synthetic hydrogels (e.g., polyethylene glycol Bjugstad et al., 2010) have provided promising results in terms of repair and regeneration after surgical implants (Nisbet et al., 2008; Green et al., 2010). Bioactive materials such as L1, growth factors, dextran or laminin, have also been used to functionalize the probe improving its tolerability (He et al., 2006; Klaver and Caplan, 2007; Azemi et al., 2011). Furthermore, cell-coated interfaces were proposed to increase the biocompatibility of the device and minimize the reaction of the host tissue (Purcell et al., 2009; Richter et al., 2011).

Although all the above-mentioned approaches may lead to potentially interesting results, the preservation of the performance of intracortical devices still remains an unresolved issue. To contribute to this challenge, we developed a strategy to improve the biocompatibility of the implant by coating microelectrodes with soft hydrogel and live cells to mimic both the mechanical properties and the biological composition of the host tissue. We used fibrin as hydrogel and either glial cells or neurons to immunologically hide the foreign object to the host tissue. By using such approach we obtained: (i) a strong adhesion of hydrogel and cells to the microelectrode without altering its electrochemical properties; (ii) a small and transient tissue reaction to the implant; and (iii) high quality of electrophysiological recordings.

## MATERIALS AND METHODS

### IMPLANTABLE WIRES

#### Quartz-coated metal microelectrodes

**FIGURE 1:**
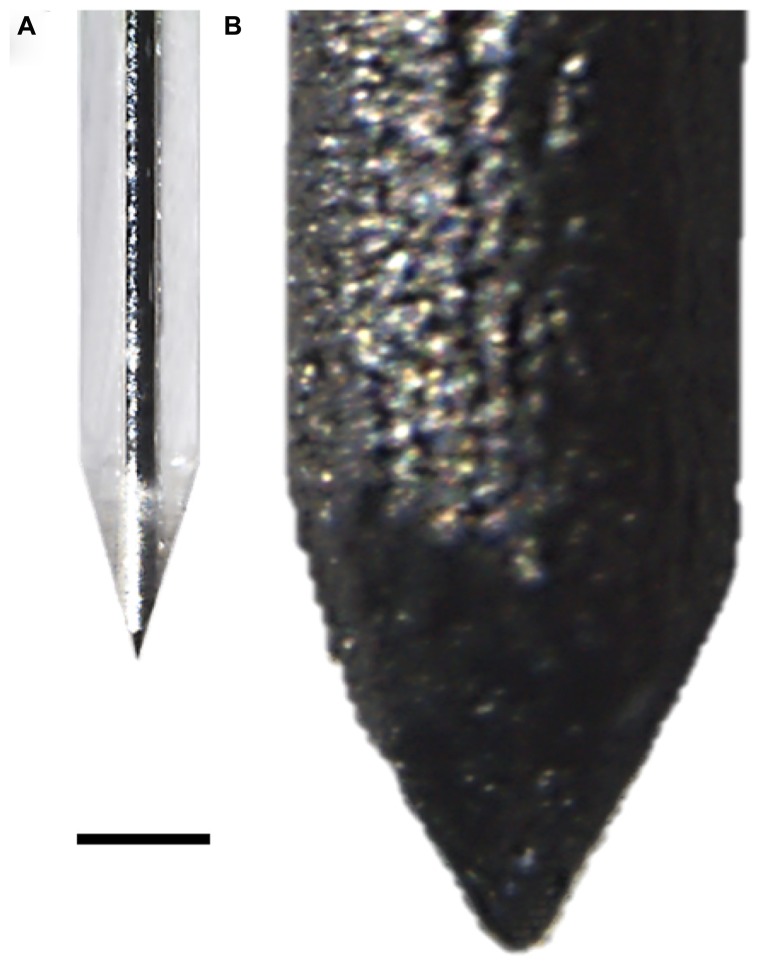
**Implantable wires used in this study. (A)** Single-core quartz insulated metal microelectrode (Thomas Recording, Giessen, Germany). **(B)** Black-lead wire. Scale bar 100 μm.

Single core quartz-insulated metal microelectrodes (Thomas Recording, Giessen, Germany) were prepared in-house by mechanically grinding 95% platinum, 5% tungsten microwires of 20 μm diameter coated with 30 μm of quartz (see **Figure 1A**). The measured impedance of these electrodes, measured in saline at 1 kHz, was typically between 500 and 700 kΩ (Ansaldo et al., 2011).

This probe was used as a seeding substrate and to evaluate the grip of the hydrogel to the electrode surface during intra-cortical insertion.

#### Black-lead sticks

Black-lead sticks were 350 μm in diameter and 3 mm long (see **Figure 1B**). The tip was mechanically ground. This probe was used to evaluate the time of hydrogel in-vivo reabsorption and the extent of tissue reaction to the implant without removing it from the brain, thanks to the possibility to cut the stick inserted in the brain with the microtome.

### FIBRIN HYDROGEL COATING

Human fibrin (TISSEEL, Baxter, Deerfield, IL, USA) from pooled human plasma was used. It was composed by two sterile components, namely the sealer protein solution (aprotinin and fibrinogen) and thrombin solution (thrombin, CaCl_2_ and factor XIII). To form a cross-linked fibrin gel, fibrinogen was dipped in the thrombin solution to generate fibrin monomers that were subsequently cross-linked by factor XIII (soluble transglutaminase). Wires were coated by alternate dippings in the two components of the fibrin sealant. The resulting electrode coating was uniform and its thickness was controllable by the number of dippings (Kim et al., 2010b). The radial thickness of the coating was evaluated by measuring the hemi-diameter of the covered probe in five points and subtracting the probe diameter. Thin films of fibrin were prepared by dropping 50 μl of the first component of the fibrin sealant and 50 μl of the second component over sterile glass slides. The drops broadened and they were left drying in air for 15 min in a laminar hood.

### EVALUATION OF THE ELECTROCHEMICAL PROPERTIES OF THE FIBRIN-COATED DEVICE

The electrochemical properties of the microelectrode were studied via electrochemical impedance spectroscopy (EIS) in a physiological aqueous solution (NaCl 0.9%). For the measurements, a sine wave (10 mV root mean square amplitude) was superimposed on the open circuit potential while varying the frequency from 1 Hz to 100 kHz. The electrochemical characterization was carried out using a potentiostat/galvanostat/ZRA (Reference 600, Gamry Instruments, Philadelphia, PA, USA) connected to a standard electrochemical cell used in a three-electrode configuration with a platinum counter-electrode (35 mm^2^ area) and an Ag/AgCl reference electrode.

### MODIFICATION OF THE MICROELECTRODE QUARTZ SURFACE

The roughness of the microelectrode quartz surface was manipulated by using an argon ion plasma etching. Sputter-coated and annealed nanometric gold islands acted as masks for quartz surface milling. The ions hit all the surface, etching both quartz and gold but where quartz was not covered, the generated holes were deeper than where it was covered by gold. The process stopped when all the gold coating was removed. Thanks to this etching, the peak-to-peak average distance was about 500 nm (Goyal et al., 2006).

### EXPERIMENTAL ANIMALS

Adult Sprague-Dawley rats (from Charles River, Calco, Italy) and Long-Evans rats (born in house) were used. All experiments were carried out in accordance with the guidelines established by the European Communities Council (Directive 2010/63/EU of September 22nd, 2010), and were approved by the Italian Ministry of Health.

#### Neurons

Neurons were isolated from E18 rat embryos. Briefly, embryos were removed, placed in cold Hank’s balanced salts solution (HBSS) and hippocampi were dissected under sterile conditions. Subsequently, the tissue was enzymatically digested with 0.125% Trypsin for 20 min at 37°C and disaggregated with a pipette. The cells were incubated with 1% Glutamax, penicillin-streptomycin, 2% B-27 supplemented Neurobasal medium in a humidified 5% CO_2_ atmosphere at 37°C. Half of the medium was changed every week. All culture media and reagents were from Invitrogen (Milano, Italy).

#### Glial cells

Glial cells were isolated from the cerebral cortex of P0 rat pups. Cortices were dissected in cold HBSS, broken into small fragments by pipetting and enzymatically digested with 0.25% Trypsin – 1 mg/ml DNase for 15 min at 37°C. After centrifugation at 1200 rpm for 5 min at 8°C, the digestion medium was removed and the pellet was suspended in 10% horse serum, penicillin-streptomycin and 33 mM glucose-supplemented minimum essential medium. Cells were plated in culture flasks and half of the medium was changed every week.

### SEEDING OF CELLS OVER THE MICROELECTRODES

Microelectrodes were fixed in a silicon cap by the end opposite to the tip and coated with 0.1% poly-D-lysine, which created a positively charged layer enhancing cell adhesion. Microelectrodes were dipped inside a vial containing the cells suspended in the medium (300000 cells/500 μl). Half of the medium was harvested from a high concentration cell culture, the so-called conditioned medium. The vials were kept in slow and continuous rotation in an incubator (2.31 rpm; relative centrifugal force 0.013 × *g*).

### EVALUATION OF THE BIOCOMPATIBILITY OF THE FIBRIN FILM

Approximately 5000 astrocytes or neurons in 100 μl of medium were plated over a thin layer of fibrin. After the cells attached to the surface, the coverslip was covered by culture medium and the cell viability was analyzed at 1, 5, 7, and 11 days. Viability was evaluated by rating the surviving cells over the total number of cells. Samples were incubated for 3 min at room temperature in Ringer-Locke solution containing propidium iodide (PI; Sigma-Aldrich, Milano, Italy; 15 μg/ml), fluorescein diacetate (FDA; Sigma–Aldrich, Milano, Italy; 5 mg/ml) and Hoechst-33342 (Sigma-Aldrich, Milano, Italy; 3.3 μg/ml). After incubation, samples were washed once in Ringer-Locke solution and immediately imaged. At least 15 different fields of view were acquired for each sample. For each field, the ratio of PI-positive cells over the total number of nuclei, identified by Hoechst-33342, was calculated (Limongi et al., 2013).

### ANIMAL SURGERY

Male Long Evans rats were anesthetized with a cocktail of Zoletil (Virbac, Carros cedex, France; 30 mg/kg) and Xylazine (Bayer, Leverkusen, Germany; 5 mg/kg) administered intraperitoneally. Their heads were then shaved and swabbed with ethanol. The anesthetized animal was placed in a stereotaxic apparatus (Myneurolab, St Louis, MO, USA). Lacrigel (Farmigea, Pisa, Italy) was distributed over the eyes to avoid dryness. An approximately 2 cm incision was made along the midline of the cranium. The underlying muscle and connective tissue were retracted to expose the skull. A craniotomy (5 mm × 3mm) was made in the parietal bone to expose the somatosensory cortex. Sterile saline was applied during pauses while drilling to help dissipate any local heating and to clean the surfaces. The exposed dura mater was wetted with saline, and carefully incised using surgical micro-scissors. Pia mater was incised too. Implantable wires were lowered perpendicularly through the cortical surface using a hydraulic microdrive (David Kopf Instruments, Tujunga, CA, USA) to 2800 μm from the surface of the brain (layer V). To minimize the variability associated with the surgery, all implants – both quartz-coated metal microelectrodes for acute experiments and black-lead sticks for chronic experiments – were performed by the same subject. During the surgery, the depth of anesthesia was monitored by testing for the absence of hindlimb withdrawal reflex and was maintained by additional doses of anesthetic (i.p. or i.m.), while the rat body temperature was kept at 36–38°C by positioning the animal onto a thermostatic heating pad.

### ACUTE NEURAL RECORDING

Acute recordings in layer V of rat cortex were performed to characterize *in vivo* the electrical performance of the fibrin-coated microelectrodes. Recordings were acquired for 20 min using a custom-made, low-power recording system (Bonfanti et al., 2008; Borghi et al., 2008) composed of a compact headstage (amplification gain = 62 dB) and a control unit for data acquisition. Raw traces were band-pass filtered between 3 Hz (first-order Butterworth) and 8 kHz (fourth-order Butterworth). The amplified signals were multiplexed, digitally converted (10-bit, 40 k sample/s) and sent via USB to a PC for subsequent analysis. LabView-based (National Instruments, Austin, TX, USA) acquisition software was used for real-time data visualization and storage. All experimental sessions were conducted inside a Faraday cage to reduce the electromagnetic noise interference.

### EVALUATION OF THE SIGNAL-TO-NOISE RATIO

To evaluate the SNR, each acquired trace was high-pass digitally filtered above 300 Hz. The signal was wavelet decomposed and thresholded to 3.5 standard deviations (SD) above and below the mean of the sample distribution to discriminate signal from noise. Waveforms were clustered using Run-Valley seeking sort method (Offline-Sorter; Plexon Inc., Dallas, TX, USA). Signal amplitude was defined as the peak-to-peak amplitude of the mean waveform for each cluster. Noise was defined as two times the calculated rms noise voltage. The SNR was calculated as follows:

(1) $$S\,N\,R=\frac{S}{2\,V\,r\,m\,s_{n}}$$

where *S* is the signal amplitude and Vrms_n_ is the voltage root mean square of the noise. SNR values were sorted into categories (Ludwig et al., 2006, 2009; Abidian et al., 2009; Kim et al., 2010b). Clusters with a SNR greater than 4 were categorized as high-quality units, while clusters with a SNR between 3 and 4 were categorized as moderate units.

### IMMUNOHISTOCHEMICAL AND IMMUNOCYTOCHEMICAL TECHNIQUES

Animals were anesthetized and transcardially perfused with cold fixative (2% paraformaldehyde, 1.25% glutaraldehyde, 2% sucrose in phosphate-buffered saline, PBS) 3, 7, and 30 days after the insertion. The brain was removed with the probe in place, fixed for an additional 24 h in the same fixative and then cryoprotected in 30% sucrose solution. Brain horizontal slices were cut (thickness, 50 μm) up to approximately 3 mm of cortical depth. Tissue sections were hydrated in PBS, blocked and permeabilized with 4% goat serum, 2% bovine serum albumin (BSA) and 0.5% Triton X-100 in PBS for 1 h with gentle rocking. Sections were then incubated overnight with primary antibodies against human fibrinogen (1:200; Dako, Milano, Italy) to visualize the presence of fibrin and glial fibrillary acidic protein (GFAP; 1:500; Sigma-Aldrich, Milano, Italy) to visualize reactive glia. The next day, sections were rinsed in PBS and incubated in secondary antibody (1:500; Invitrogen, Milano, Italy) for 3 h. All sections were mounted with ProLong^TM^ Gold Antifade reagent with DAPI (Invitrogen, Milano, Italy) to stain nuclei.

Cultured cells were fixed in cold fixative (2% paraformaldehyde, PFA, 1.25% glutaraldehyde, 2% sucrose in PBS) for 20 min. Cell-coated substrates were rinsed in cold PBS, blocked and permeabilized with 10% goat serum, 2% BSA and 0.5% Triton X-100 in PBS for 1 h with gentle rocking agitation. Samples were later incubated for 90 min with the following primary antibodies in blocking solution: anti-GFAP (1:500; Sigma-Aldrich, Milano, Italy), anti-NeuN (1:500; Millipore, Milano, Italy) and anti-βIII tubulin (1:500; Sigma-Aldrich, Milano, Italy). The seeded microelectrodes were rinsed in PBS and then incubated for 3 h with Alexa-Fluor 633 goat anti-rabbit and Alexa Fluor 488 goat anti-mouse (1:500; Invitrogen, Milano, Italy). All samples were mounted with ProLong^TM^ Gold Antifade reagent with DAPI (Invitrogen, Milano, Italy).

### QUANTITATIVE IMMUNOFLUORESCENCE ANALYSES

Images were acquired using an Olympus BX51 fluorescence microscope (Melville, NY, USA) equipped with X-Cite 120 fluorescence illumination system (EXFO, Canada, USA) and with a color digital camera (MBF Bioscience, Williston, VT, USA). Images for the specific antibody were taken by keeping the same exposure time and gain. Fluorescence intensity profiles were obtained by using the custom written MATLAB program MINUTE (Microelectrode INterface Universal Tool for Evaluation; Potter et al., 2012). The mean intensity of fluorescence at discrete distances was compared by one-way repeated measures ANOVA followed by the Bonferroni’s *post hoc* test when more than two groups were involved. Significance for all tests was determined at *P* < 0.05.

## RESULTS

### ELECTROCHEMICAL PROPERTIES AND QUALITY OF THE RECORDINGS OF FIBRIN-COATED MICROELECTRODES

**FIGURE 2:**
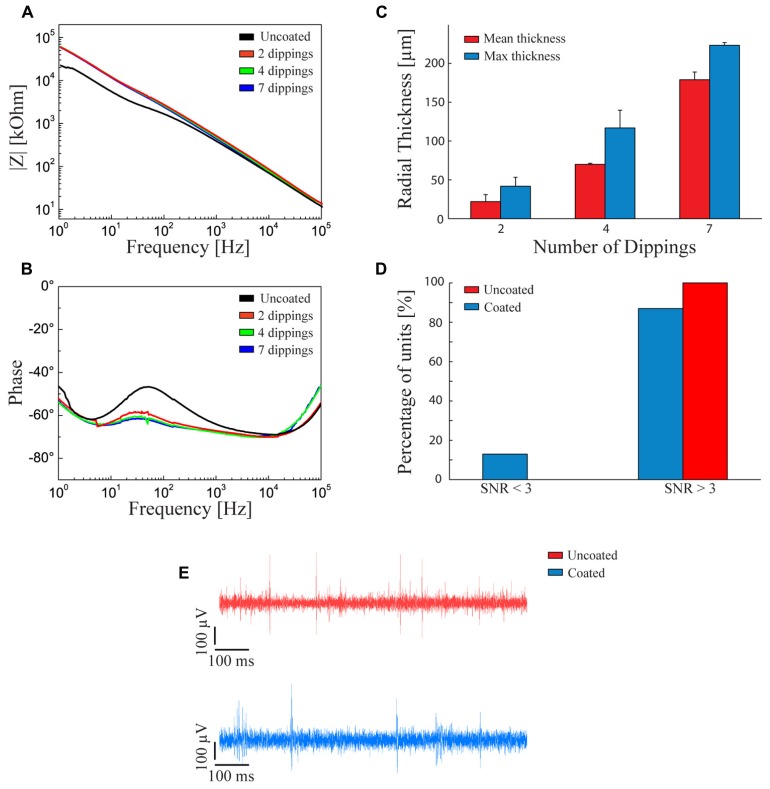
**Fibrin coated microelectrodes. (A,B)** Electrochemical impedance spectroscopy. Impedance modulus **(A)** and phase **(B)** with respect to the frequency between 1 Hz and 100 kHz. The black line refers to the bare microelectrode. The red, green, and blue lines represent the behavior of fibrin-coated microelectrodes dipped two, four, and seven times into the two sealant components, respectively. **(C)** Thickness of the swollen fibrin coating deposited by two, four, and seven dippings into the two sealant components. Red and blue bars represent the mean and maximum (±SD) radial thickness of the coating, respectively. The procedure with four dippings allowed getting close to the optimal radial thickness of 100 μm, a critical value for the survival of underlying cells and the quality of neural recordings. **(D)** Signal-to-noise ratio in the recordings. Percentage of units with SNR < 3 (poor units), SNR > 3 (moderate or quality units) recorded with uncoated (red bars) and fibrin-coated (blue bars) microelectrodes. **(E)** Examples of recording traces with uncoated (red) and fibrin-coated (blue) microelectrodes. **(D,E)** The measurements were taken immediately after surgery.

We analyzed the effect of the fibrin layer on the electrical properties of the coated electrode. Coating thickness was progressively increased by dipping the electrode in both fibrinogen and thrombin from 2 to 7 times. **Figure 2A** shows that increasing the fibrin sheath did not significantly affect the electrochemical properties of the microelectrode at 1 kHz, that is the fundamental frequency of neuronal action potentials (Williams et al., 2007) both in terms of impedance modulus (**Figure 2A**) and impedance phase (**Figure 2B**). The lack of alteration of the electrical properties of the electrodes is likely due to the swelling of the hydrogel and to the associated absorption of water and ions from the saline solution that maintain an optimal conductivity (Kim et al., 2004a).

However, once the electrode is implanted in-vivo, the swelling of the fibrin hydrogel coating increases the distance between the tissue and the electrode. Under our conditions, the fibrin coating displayed a smaller swelling than other types of hydrogels, such as alginate (Kong et al., 2004; Kim et al., 2010b) and was controllable in thickness.

To obtain the desired thickness of coating, the following constraints had to be considered: (i) the hydrogel thickness should be less than 100 μm to avoid oxygen deprivation of cells seeded over the probe (Kim et al., 2004a); (ii) the thicker the hydrogel layer, the smaller the gap between the Young modulus of the device and that of the host tissue at the interface; (iii) the thicker the coating, the greater the damage of the host tissue; and, finally, (iv) the swollen hydrogel layer should be thinner than 140 μm to allow activity recordings with high SNR (Henze et al., 2000). According to this evaluation, we decided to adopt a thickness of about 100 μm after swelling. **Figure 2C** shows the relationships between the number of dippings of the electrode in the two components of the fibrin sealant and the average and maximum thickness of the coating. Four dippings were found to be optimal to obtain the desired hydrogel thickness.

Recording capabilities of the coated versus non-coated electrodes were evaluated by calculating the SNR of neural waveforms recorded from rat cerebral cortex (see Methods). 85% of the waveforms recorded with fibrin-coated electrodes showed a SNR > 3. This performance is comparable (no statistically significant difference) to that of non-coated microelectrodes (**Figures 2D,E**).

### BIOCOMPATIBILITY OF THE FIBRIN LAYER

**FIGURE 3:**
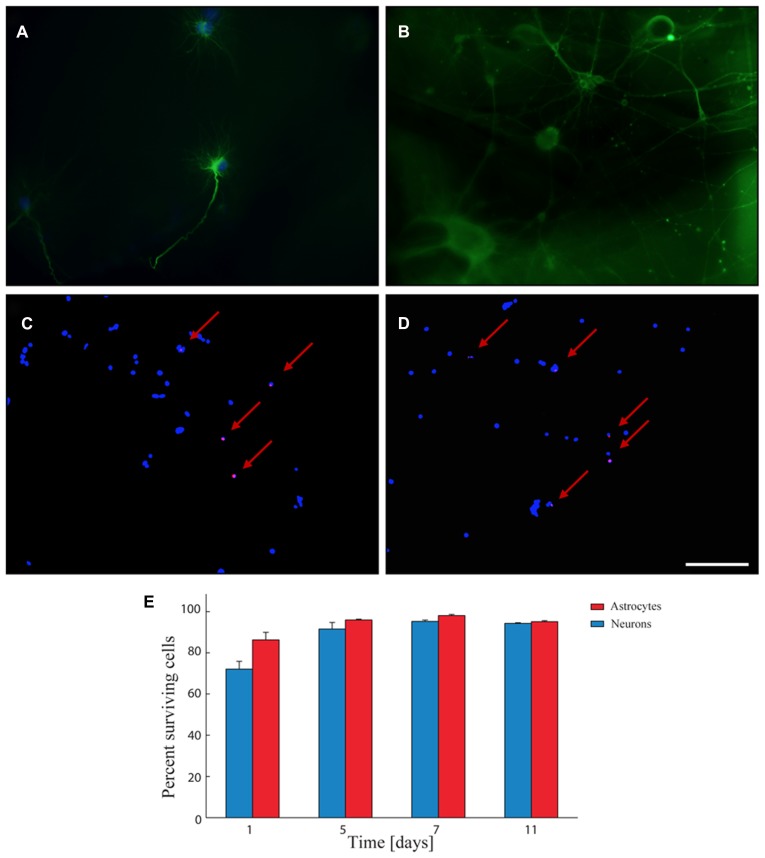
**Biocompatibility of the fibrin layer. (A,B)** Representative immunofluorescence image of glial cells in fibrin layer **(A)** stained for GFAP (green) and DAPI (blue) and of primary neurons in fibrin layer stained for βIII-tubulin (green; **B**). **(C,D)** Representative images of glial cells **(C)** and primary neurons **(D)** grown in fibrin layer stained with Hoechst (blue-stained nuclei) and propidium iodide (red-stained nuclei). Red arrows indicate the PI-positive nuclei. Scale bar: 50 μm in **A**; 100 μm in **B–D**. **(E)** Quantitative analysis of the viability of primary neurons (blue bars) and astrocytes (red bars) cultured in the presence of fibrin at 1, 5, 7, and 11 days *in vitro*. The percentages of surviving cells (means ± SD) were calculated based on the ratio of total (Hoechst-positive) nuclei minus PI-positive nuclei divided by the total nuclei. Viability values increase and approach the 100% 7 days after seeding.

A viability assay was used to assess the biocompatibility of the fibrin hydrogel deposited as a thin film for primary neurons and glial cells seeded onto the sample. Both astrocytes (**Figure 3A**) and primary neurons (**Figure 3B**) grew well onto the fibrin layer. The assay (**Figures 3C,D**) demonstrated that the viability of both types of cells over time in culture was comparable although, one day after seeding, the mortality of neurons was slightly higher than that of glial cells (86% of surviving glial cells versus 72% of surviving neurons; **Figure 3E**). Viability progressively increased up to 7 days in culture (98% of glial cells versus 95% of neurons; **Figure 3E**) and remained high up to 11 days in culture (95% of glial cells versus 94% of neurons; **Figure 3E**). The extent of viability was in the same range of that obtained under standard conditions in the absence of the fibrin layer (Ghezzi et al., 2011).

### MODIFICATION OF THE MICROELECTRODE SURFACE

**FIGURE 4:**
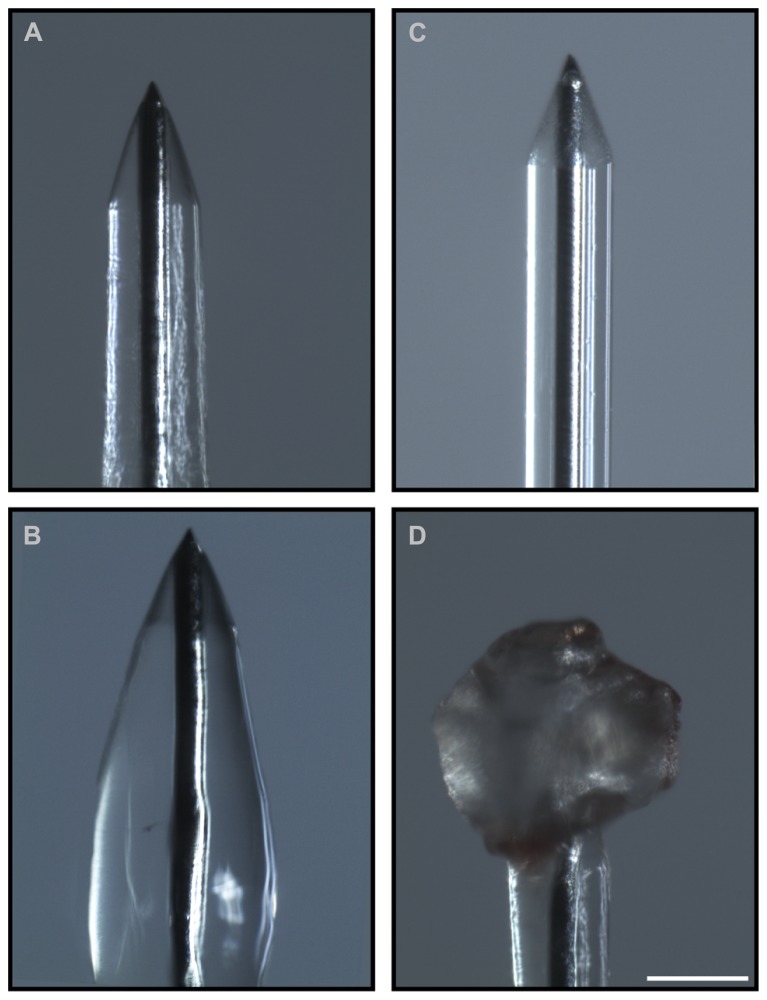
**Adhesion of the hydrogel to the microelectrode during the surgical procedure**. Optical images of the coated microelectrodes before **(A,B)** and after **(C,D)** the insertion in the brain. Fibrin coating did not resist the shear stress during insertion through the dura mater and detached from the electrode surface in 90% of cases (60% of cases after removal of the dura mater). **(C,D)** Representative images of the adhesion of the hydrogel to the microelectrode before the treatment that increases the microelectrode roughness: during insertion the coating was entirely peeled **(C)** or partially piled up on the tip **(D)**. Scale bar 100 μm.

**FIGURE 5:**
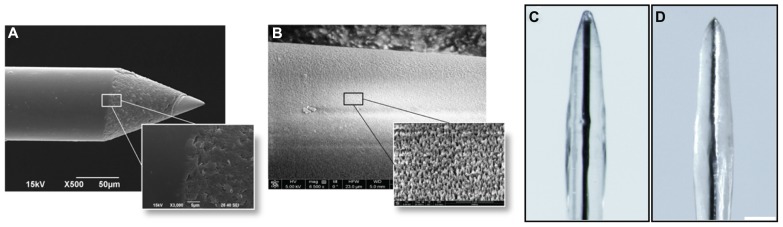
**Increase in roughness of the microelectrode surface**. **(A)** Scanning electron micrographs of the microelectrodes before the etching treatment. **(B)** Scanning electron micrographs of the microelectrodes after the treatment. Scale bar 50 μm. The obtained peak-to-peak average distance was about 500 nm. **(C,D)** Microelectrodes with increased surface roughness are imaged before **(C)** and after **(D)** the insertion in the cerebral cortex. Coated nanostructured microelectrodes could be effectively inserted in the cerebral cortex deprived of dura mater without any noticeable damage or shift of the coating in the 100% of cases. Scale bar 100 μm.

The results of the preliminary experiments indicate that the fibrin coating seems to be ideal both in terms of modulation of the coat thickness and biocompatibility for primary cells. However, smoothness of the electrode quartz surface somehow prevented a tight anchorage of the hydrogel to the electrode surface. Thus, during implantation of the electrode into the brain tissue, the fibrin coating (**Figures 4A,B**) could not resist to the shear stress during insertion through the dura mater and detached from the electrode surface in 90% of the cases (**Figures 4C,D**). Also, after the removal of the dura mater, the hydrogel stayed attached to the microelectrode surface only in 40% of the penetrations. As these initial results were poorly reproducible and not sufficiently satisfactory, we etched the quartz surface of the microelectrode to increase its roughness and thereby the grip of the hydrogel to the surface (**Figures 5A,B**). After this treatment, microscopic inspections demonstrated that the fibrin layer was still firmly adherent to the quartz surface in the 100% of cases when the coated microelectrode was inserted and extracted from the somato-sensory cortex deprived of dura mater (**Figures 5C,D**).

### *IN-VIVO* REABSORPTION OF THE HYDROGEL AND TISSUE REACTION TO THE COATED ELECTRODE

To evaluate the time of reabsorption of the hydrogel in-vivo and the related tissue reaction, it was necessary to extract the implanted electrode before sectioning. However, during extraction of the implant from the perfused brain, part of the tissue could remain attached to the implant and be distorted or removed together with the probe, thus altering the evaluation of the electrode coating and of the surrounding tissue. To circumvent these problems, we implanted graphite sticks (black-lead rods) that, although thicker than quartz/tungsten microelectrodes, can be easily cut in the microtome, thus allowing to avoid their extraction from the brain before histochemical examination.

**FIGURE 6:**
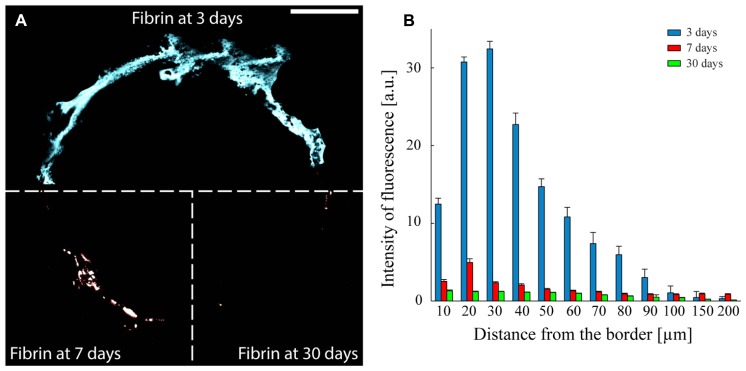
**Fibrin reabsorption after implantation. (A)** Representative images to illustrate the presence of the fibrin over time. The three sectors of the panel represent the three analyzed implant times: 3, 7, and 30 days. Scale bar 100 μm. **(B)** Quantitative evaluation of fibrin immunoreactivity (a.u., arbitrary units; means ± SEM) as a function of distance from the tissue-implant interface after 3 (blue bars), 7 (red bars), and 30 (green bars) days from surgery. An almost complete dissolution of the fibrin hydrogel is already observed after 7 days *in vivo*. One-way repeated measures ANOVA followed by the Bonferroni *post hoc* test; *P* < 0.05 the intensity of fibrin immunoreactivity at 7 or 30 days *versus* 3 days in the distance range 10–90 μm from the implant-tissue interface (*n* = 41, 52, and 58 slices for 3, 7, and 30 days, respectively, from three implants/time point).

Fibrin reabsorption was determined by immunofluorescence after 3, 7, and 30 days from implant (**Figure 6A**). Quantitative analysis of fibrin immunoreactivity as a function of time and distance from the electrode edge (**Figure 6B**) revealed that the intensity of fluorescence steadily decreased in the days immediately after the insertion and that the coating was almost completely reabsorbed, in agreement with previous reports (de Vries et al., 2002). The intensity of the fibrin marker was significantly lower already 7 days after the insertion compared to the values obtained after 3 days, at any distance from the microelectrode edge, up to 90 μm (*P* < 0.05 after 7 days from the insertion; one-way repeated measures ANOVA followed by the Bonferroni’s *post hoc* test), while no differences in the extent of fibrin reabsorption were present between 7 and 30 days up to 90 μm from the brain-microelectrode interface.

**FIGURE 7:**
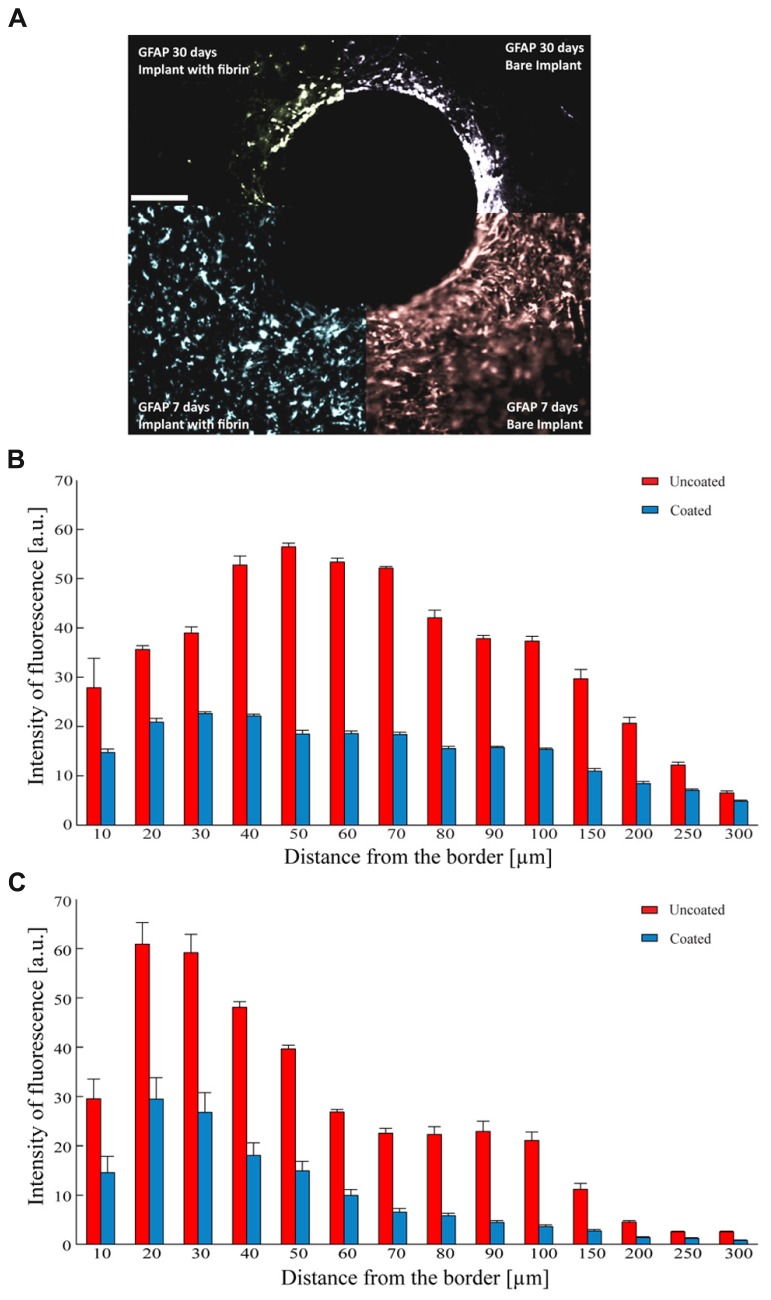
**Astrocyte reaction to the implant. (A)** Representative images of GFAP immunoreactivity detected 7 (lower quadrants) and 30 days (upper quadrants) after the implant of uncoated (right quadrants) and fibrin-coated (left quadrants) black-lead wires. Scale bar 100 μm. **(B,C)** Analysis of GFAP-positive reactive astrocytes as a function of the distance from the tissue-implant interface. Mean (±SEM) intensity of GFAP immunoreactivity (a.u.) at 7 **(B)** and 30 **(C)** days from the implant. Red and blue bars refer to uncoated and fibrin-coated microelectrodes, respectively. One-way repeated measures ANOVA; *P* < 0.05 coated *versus* uncoated electrodes at all distances for both 7 and 30 days post-implant (*n* = 54 and 58 slices for 7 and 30 days, respectively from 3 implants/time point).

As astrocytes play a dominant role in the tissue reaction and in the formation of the glial scar, we investigated the glial reaction by monitoring the expression of GFAP around the implantation site. The implant of a bare electrode elicited a strong astrocytic reaction as determined 7 days after the insertion, that became denser and thinner over time, leading to a full encapsulation of the electrode (**Figure 7A**). In contrast, the implant of a fibrin-coated electrode elicited a weaker astrocyte reaction at 7 days, which further decreased to very low levels after 30 days (**Figure 7A**). Quantification of the immunostaining after 7 (**Figure 7B**) and 30 (**Figure 7C**) days as a function of the distance from the electrode-tissue interface revealed that GFAP expression was markedly more pronounced in response to the implant of a bare electrode than that of a fibrin-coated one at both 7 and 30 days post-implant at any distance from the brain-microelectrode interface (*P* < 0.05 after 7 or 30 days of implant; one-way repeated measures ANOVA). In the case of a bare electrode, the peak of GFAP immunoreactivity, localized at 50 μm from the wire-host tissue interface at 7 days became more intense and moved closer to the electrode edge at 30 days (distance: 30 μm). In contrast, in the case of fibrin-coated microelectrodes, the astrocyte reaction in the acute phase (7 days) was milder and evenly distributed around the implant, extending up to 250 μm, while it strongly decreased with time being barely detectable only for 90 μm from the electrode at 30 days.

### COATING MICROELECTRODES WITH CELLS AND FIBRIN HYDROGEL

**FIGURE 8:**
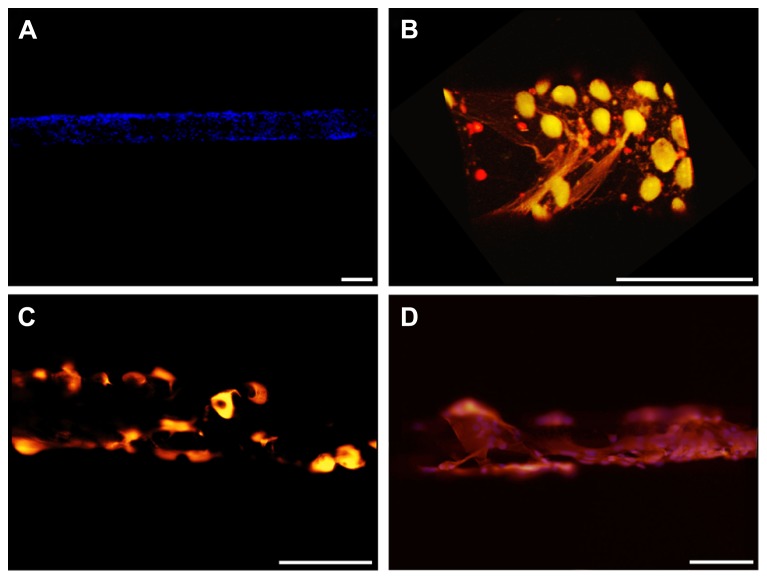
**Cell-coated microelectrodes. (A)** Representative fluorescent image illustrating the layer of cells adhered to the microelectrode. DAPI staining (blue) reveals cell nuclei. **(B)** Three-dimensional reconstruction of a representative microelectrode coated with neurons. Cells were stained for NeuN (green) and βIII-tubulin (red). **(C,D)** Representative fluorescent images of astrocytes grown on the microelectrode. Cells were stained for GFAP (red) and DAPI (blue). Scale bars 100 μm.

We next attempted to seed and grow live cells on the electrode surface with the aim of making the artificial device well tolerated by the host tissue after the reabsorption of the fibrin layer. Both primary neurons and glial cells were seeded. The cells seeded with the method illustrated in this paper grew vigorously and formed a uniform compact monolayer on the curved surface of the electrode (**Figure 8A**). Specific staining of primary neurons with either NeuN or βIII-tubulin (**Figure 8B**) and of astrocytes with GFAP (**Figures 8C,D**) revealed that, using conditioned medium, both types of primary cells can grow on the device.

**FIGURE 9:**
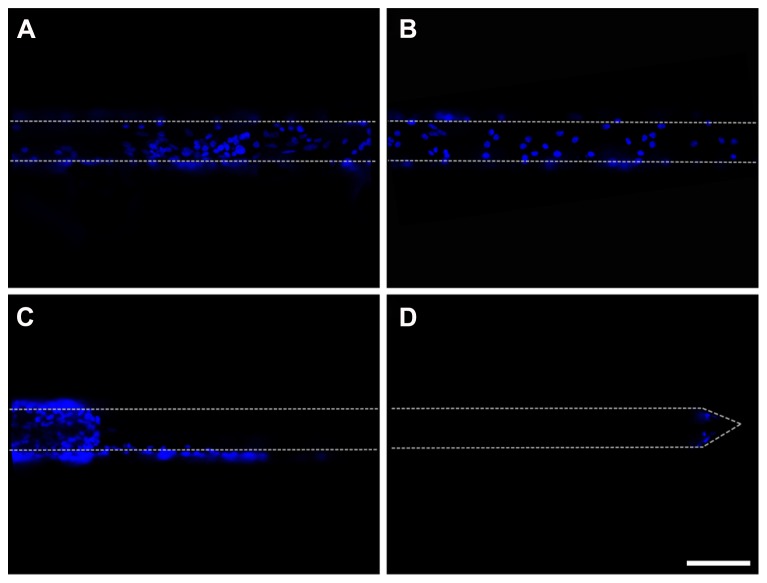
**Insertion of cell-coated microelectrodes. (A,B)** Representative fluorescent images illustrating the layer of glial cells adhered to a microelectrode. DAPI staining (blue) reveals cell nuclei. The cell-coated microelectrode was inserted 2 mm in depth and the images were acquired from the non-inserted segment of the probe. **(C,D)** Representative fluorescent images of the same microelectrode, illustrating the effects of the insertion of the probe on the adhered glial cells (images acquired along the wire from the inserted segment of the wire). DAPI staining (blue) reveals cell nuclei. Scale bar 100 μm. Dot lines point out the microelectrode shape.

**FIGURE 10:**
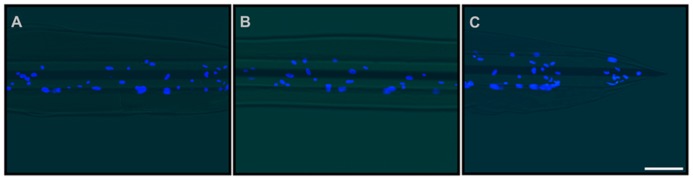
**Double-coating of microelectrodes with astrocytes and fibrin hydrogel**. Representative images of the final prototype of the bio-inspired hybrid microelectrode: 3 mm far from the tip **(A)**, 1.5 mm far from the tip **(B)** and the tip **(C)**. Fluorescence images (DAPI staining of neuronal nuclei) were merged with the optical image of the microelectrode. The images revealed the microelectrode structure, the fibrin hydrogel sheath surrounding it and the layer of glial cells embedded within the hydrogel. Scale bar 100 μm.

Cells adhered to the microelectrodes were partially stripped during device insertion into the brain (**Figures 9A–D**). Richter et al. (2011) quantified the effects due to the pulling force acting onto a cell layer and demonstrated the abrasion of the adhered cells and a protective role played by fibrin coating. We therefore decided to protect the cell layer by embedding the cells into a fibrin layer which could also increase the mechanical compliance of the inserted microelectrode (**Figures 10A–C**).

**FIGURE 11:**
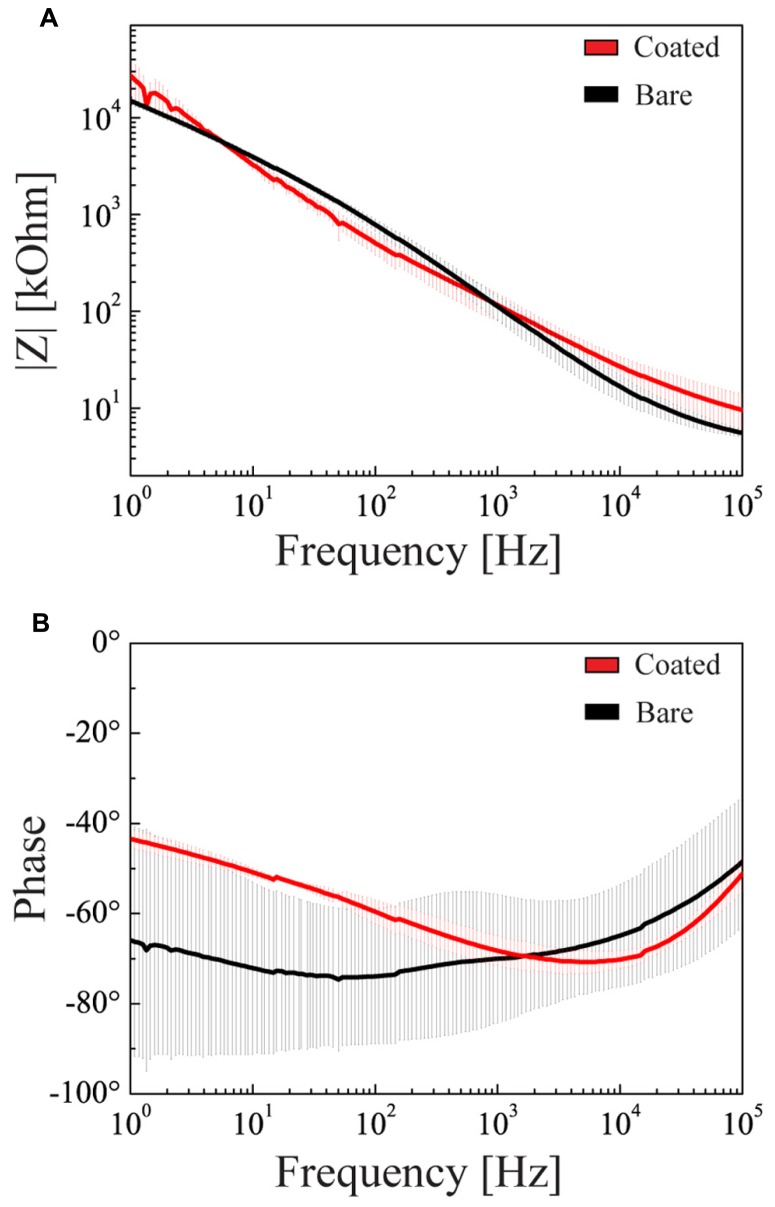
**Electrochemical properties of double-coated microelectrodes**. Impedance modulus **(A)** and phase **(B)** as a function of the frequency in the 1 Hz–100 kHz range (mean ± SD). Black and red lines refer to the bare microelectrode and to the microelectrode coated with glial cells and hydrogel, respectively.

Although previous experiments demonstrated that hydrogel coating does not affect the electrochemical properties of the microelectrodes, we repeated these measures with the hybrid cell/hydrogel-coated electrodes, as the cell layer covering the device could alter the current passage. We demonstrated that the combined coating did not significantly affect either the impedance modulus or phase at 1 kHz with respect to uncoated or fibrin-coated electrodes (**Figures 11A,B**).

## DISCUSSION

The aim of this study was to realize a bio-inspired hybrid device. We developed a strategy to improve the biocompatibility of microelectrodes mimicking the properties of the host tissue in order to increase the biocompatibility over time. To this aim, we modified the device surface to decrease the tissue reaction against the implant and thus increase the working life of the intra-cortical implant. The two main goals we achieved were: (i) to minimize the mechanical difference between the rigid microelectrode shaft and the soft and expanding-retracting brain tissue and (ii) to coat the electrode with a live and tissue-friendly autologous cell layer to improve the tissue tolerance to the foreign body. These aims were obtained by coating the microelectrode with a soft layer of fibrin hydrogel and by coupling it with a layer of living cells (either astrocytes or neurons). While both coatings did not alter the electrochemical properties of the electrode, the combination of the two seems particularly advantageous. In fact, the hydrogel minimizes the continuous trauma due to the different compliance of the electrode and the brain tissue and, at the same time, protects the cell layer from the stress due to the device insertion inside the brain. This can prevent the typical foreign body-dependent inflammation that ends up with a glial scar encapsulating and electrically insulating the device.

The thickness of the soft coating turned out to be a key point. In fact, while the compliance increases with the thickness of the hydrogel, the coating should not be too thick to allow for oxygen supply to the cells seeded on the probe and reliable extracellular recordings of spike activity over the background noise. Another important point is that hydrogels swell when immersed in a saline solution such as the extracellular fluid, and swelling *per se* can compress the surrounding tissue and increase the distance between the brain and the recording site. To comply with these requirements, we exploited fibrin as a hydrogel for the following reasons: (i) coating thickness can be precisely controlled by the number of dippings during the deposition procedure; (ii) it does not affect the electrochemical properties of the microelectrode; (iii) it allows a good quality of recordings (85% of the fibrin-coated electrodes recorded high-quality brain signals with SNR > 3); and (iv) its swelling is moderate with respect to other types of hydrogel (i.e., alginate swelling is such that the recording from target neurons is prevented; Kim et al., 2010b).

Biocompatibility of the fibrin hydrogel was also an important issue. We demonstrated that fibrin coating markedly reduces the extent of astrocyte reaction at both 7 and 30 days after the implant, compared to the bare wire. The astrocyte activation following the implantation of coated wires is more diffuse and less intense, yielding to a significant lesser extent of glial scar and device encapsulation.

Another advantage of the fibrin hydrogel besides its biocompatibility is the progressive, almost complete enzymatic degradation by the host tissue over time. While the hydrogel thickness is an important issue to prevent acute reactions, the progressive degradation does not pose limitations for chronic recordings. While the soft fibrin layer is reabsorbed, cells seeded onto the electrode surface might have integrated with the host tissue both by exchange of extracellular messengers (such as growth factors or neurotransmitters) and by growth of processes and establishment of new connections. Thus, after the disappearance of the fibrin hydrogel, the electrode could already be interfaced and well tolerated by the host tissue.

Another important point was to verify the integrity of the coating upon insertion of the device into the tissue. The shear stress of insertion not only had critical consequences on the unprotected cell coating of the electrode, but was also effective, in a large percentage of cases, in stripping the fibrin hydrogel from the smooth surface of the electrode. We set-up a treatment to increase the roughness of the quartz electrode surface, achieving a better grip for both the cell layer and the fibrin sheath.

Our study has demonstrated the possibility to produce tissue-friendly microelectrodes well-tolerated and functional over time. This is fundamental to realize electrodes for chronic recordings. Additionally, the use of materials that can be withdrawn directly from the individual patient before the implant is a fundamental approach to reduce the immunoreaction to the minimum. Our solution, using fibrin and cells fulfills both these requirements. The fibrin precursor, fibrinogen, can be easily extracted from the patient’s blood. Neurons and glial cells can be reprogrammed from patient’s fibroblasts either from iPS cell clones or, more safely, by direct reprogramming. The latter procedure has been successfully obtained for dopaminergic neurons (Broccoli et al., 2011; Caiazzo et al., 2011).

## CONCLUSIONS

The properties of the tissue-electrode interface, including electrical and mechanical integration, are crucial in determining long-term reliability and functionality of neural implants. The major problems of chronic neural prostheses are the immune and inflammatory tissue responses that occur after device implantation. This causes a significant increase of the electric impedance because of the growth of a dense fibrous sheath. Additionally, target neurons are pushed away by the scar so that the electrode becomes isolated from the surrounding tissue.

In this work, we developed a new method to control the biological response to electrode implantation and promote a more efficient integration of implanted microelectrodes. We developed a bio-inspired hybrid probe obtained with the seeding of a compact sheath of cells encapsulated in a thin re-absorbable fibrin layer and demonstrated that such a device is biocompatible, does not alter the electrochemical properties of the electrode, allows recordings of good quality and markedly reduces the tissue inflammatory reaction. This device has therefore a good potential to improve the electrical integration at the neuron-electrode interface and support the long-term success of neural prostheses.

## Conflict of Interest Statement

The authors declare that the research was conducted in the absence of any commercial or financial relationships that could be construed as a potential conflict of interest.
